# Epithelial–Mesenchymal Transition in Non-Small Cell Lung Cancer Management: Opportunities and Challenges

**DOI:** 10.3390/biom14121523

**Published:** 2024-11-28

**Authors:** Yunyao Ye, Shanxun Yu, Ting Guo, Sihui Zhang, Xiaozhou Shen, Gaohua Han

**Affiliations:** 1 Department of Oncology, Taizhou People’s Hospital Affiliated to Nanjing Medical University, Taizhou 225300, China; yeyunyao20230419@njmu.edu.cn (Y.Y.); 15720803282@163.com (S.Y.); zhangshtaizhou@163.com (S.Z.); kevinsxz@163.com (X.S.); 2 Central Lab, Taizhou People’s Hospital Affiliated to Nanjing Medical University, Taizhou 225300, China; guoting20230419@njmu.edu.cn

**Keywords:** epithelial–mesenchymal transition, metastasis, tumor immune microenvironment, non-small cell lung cancer, immune checkpoint inhibitor

## Abstract

Lung cancer, the leading cause of death worldwide, is associated with the highest morbidity. Non-small cell lung cancer (NSCLC) accounts for 80–85% of lung cancer cases. Advances in the domain of cancer treatment have improved the prognosis and quality of life of patients with metastatic NSCLC. Nevertheless, tumor progression or metastasis owing to treatment failure caused by primary or secondary drug resistance remains the cause of death in the majority of cases. Epithelial–mesenchymal transition (EMT), a vital biological process wherein epithelial cancer cells lose their inherent adhesion and transform into more invasive mesenchymal-like cells, acts as a powerful engine driving tumor metastasis. EMT can also induce immunosuppression in the tumor environment, thereby promoting cancer development and poor prognosis among patients with NSCLC. This review aims to elucidate the effect of EMT on metastasis and the tumor immune microenvironment. Furthermore, it explores the possible roles of EMT inhibition in improving the treatment efficacy of NSCLC. Targeting EMT may be an ideal mechanism to inhibit tumor growth and progression at multiple steps.

## 1. Introduction

Non-small cell lung cancer (NSCLC) is associated with the highest morbidity and mortality among all types of cancer [1] and, therefore, is a threat to human health. The majority of patients with NSCLC present with an advanced stage of the disease along with lymph node invasion and distant metastasis at the time of diagnosis; consequently, the surgical options are limited. The five-year survival rate of these patients is <10% [2]. Metastasis accounts for 90% of cancer-related mortality [3]. Epithelial–mesenchymal transition (EMT) is the first domino in the multi-step invasion–metastasis cascade of cancer cells from leaving the primary site to forming metastatic colonies at distant organs [4]. The “seed and soil” theory, proposed by Stephen Paget, describes the malignant biological behavior of cancer [5]. The disseminating tumor cells and tumor microenvironment (TME) correspond to the seeds and soil, respectively. TME is defined as the environment around the tumor that hosts the tumor cells, comprising immune cells, stromal cells, fibroblasts, endothelial cells, the extracellular matrix (ECM), and various signaling molecules [6]. Stephen Paget hypothesized that the right seeds could only survive in appropriate soil as “when a plant goes to seed, its seeds are carried in all directions; but they can only live and grow if they fall on congenial soil” [5]. This hypothesis partially explains the phenomenon of some types of cancer favoring metastasis to certain organs, such as brain and bone metastases from lung cancer. The disseminating tumor cells, TME, and their interaction play a crucial role in facilitating metastasis. The cancer cells metastasize to a site-specific organ following EMT at the primary site, invade nearby normal tissue parenchyma, infiltrate into the vessels, disseminate to distant target tissues via blood vessels, and facilitate the extravasation of cancer cells into the parenchyma of these tissues [7]. The future course of metastases, i.e., the elimination, dormancy, or formation of a metastatic lesion, depends on the interaction between the specific TME and cancer cells.

The introduction of immune-checkpoint inhibitors (ICIs) has improved the survival and quality of life of patients with NSCLC, thereby becoming milestones in the field of tumor treatment. Immunotherapy has facilitated the achievement of favorable treatment outcomes in patients with NSCLC. However, the effectiveness of immunotherapy varies greatly in different patient subgroups; moreover, patients with multiple metastatic sites may exhibit a heterogenous response, i.e., the cells at some metastatic sites may respond, whereas those at other sites continue to progress. This phenomenon is referred to as “differential response” in clinical practice [8]. In addition, similar to other treatment responses, the majority of patients undergoing immunotherapy develop drug resistance, which manifests as no response (innate resistance) or no response after the initial response (acquired resistance). EMT endows cancer cells with plasticity and heterogeneity, contributing to differential treatment response. EMT has also received increased attention owing to its role in inducing immunosuppression by affecting cancer cells and TME immune cells and components [9] to promote tumor progression and treatment failure.

This review summarizes the molecular mechanisms involved in EMT that affect tumor progression and immunotherapy outcomes in patients with NSCLC. Furthermore, this review explores the opportunities to improve the prognosis of patients of NSCLC via targeting EMT.

## 2. EMT

EMT is a complex cellular process wherein cells lose their epithelial characteristics and gradually develop mesenchymal-like characteristics. The phenotypic transformation involves the weakening of the cell–cell junctions, apical–basal polarity, interactions with the basement membrane, and enhanced cell motility. Betty Hay first proposed the concept of epithelial-to-mesenchymal transformation in 1967 while studying embryogenes [10]. This term was replaced by “epithelial-to-mesenchymal transition” in 2003 at the first meeting of The Epithelial–Mesenchymal Transition International Association (TEMTIA) to facilitate the accurate visualization of the dynamic and transitional process [10,11]. EMT involves various physiological and pathological events, such as embryonic development, tissue regeneration, and wound healing, along with organ fibrosis and cancer progression [11]. EMT is a reversible process, and mesenchymal cells can revert to the epithelial state by undergoing mesenchymal–epithelial transition. The presence of interstitial markers and the absence of epithelial markers have been used to characterize the occurrence of EMT in most previous studies. E-cadherin and cytokeratins have been used as epithelial markers, whereas N-cadherin and Vimentin have been commonly used as mesenchymal markers [12].

EMT is a physiologically and pathologically partial or incomplete process in most cases in vivo [13]. Moreover, the transition of epithelial cells into mesenchymal cells results in different intermediate states according to the different EMT signals. The “metastable state” refers to the existence of intermediate hybrid epithelial and mesenchymal phenotypes [14]. Cells lose their apico–basal polarity and cell–cell adhesions and gain front–back polarity and enhanced cell motility in steps during E to M transition. The phenotype spectrum is not a linear continuous transformation, and a clear boundary cannot be distinguished between different intermediate states [15]. Cells can exhibit more interstitial manifestations or more epithelial phenotypes in response to external signals owing to the reversibility of the process; thus, it is difficult to determine whether there is a “point of no return” in the EMT process [15]. Consequently, EMT endows cells with strong plasticity and heterogeneity. Epithelial–mesenchymal plasticity is defined as the ability of the cell to move between different states of E to M or reversal transition [16]. Huang et al. analyzed 43 ovarian cancer cell lines after gaining mesenchymal characteristics and reported that only 50% of cells with an intermediate phenotype (co-expressing cytokeratin and vimentin) induced N-cadherin expression [17]. This heterogeneity of the EMT phenotype suggests that relying solely on some specific epithelial or stromal markers to characterize the occurrence of EMT is not sufficient. Epithelial and stromal gene expression profiles are background specific; however, E-cadherin plays a critical role in maintaining epithelial integrity in physiological and pathological settings [18]. The suppression of E-cadherin is a crucial step in EMT. Snails are the first direct repressors of E-cadherin expression to be described [19]. Therefore, classical studies on EMT regulation investigated the inhibitory effect of EMT transcription factors (EMT-TFs), such as the SNAIL, ZEB, and TWIST families, on E-cadherin expression. EMT activation pathways include the WNT, NOTCH, TGFβ, PI3K-Akt, and JAK-STAT pathways. These pathways activate the expression of EMT-TF to induce changes in the epithelial- or stromal-related gene expression profiles, thereby regulating cell proliferation, migration, and other related phenotypic changes. Transforming growth factor-β (TGFβ) is a critical EMT inducer that binds to the complexes of TGFβ receptor type 1 (TGFβ R1) and TGFβ R2, leading to the phosphorylation of SMAD2 and SMAD3 [20], which form complexes with SMAD4. These trimeric SMAD complexes induce the upregulation of transcriptional factors, such as Snail, Slug and Zeb1, Zeb2/SIP1, and Twist. These proteins migrate to the nucleus and bind to E-box elements in the promoter region of the gene encoding the protein E-cadherin. They subsequently recruit histone deacetylases (HDACs) and other co-repressors to facilitate chromatin condensation and the transcriptional suppression of E-cadherin expression [21]. EMT-TF also upregulates the expression of TGFβ ligands. This establishes an autocrine signal that creates a positive feedback loop that helps maintain EMT activation. In addition, the co-expression of SNAIL and SLUG increases the expression of the TGFβ pathway genes [22]. These effects lead to the activation and maintenance of EMT effects. The Wnt signaling pathway is an evolutionarily conserved pathway that plays an important role in the development and maintenance of tissue homeostasis across species [20]. The Wnt pathway has a multi-effect biological function, i.e., it influences cell proliferation, differentiation, and maturation. Consequently, it has become a hot topic in the field of stem cell biology and cancer research. The activation of the canonical Wnt pathway induces β-catenin translocation into the nucleus, leading to the formation of transcription factor complexes containing TCF/LEF via Wnt ligands binding to the Frizzled receptor family on the cell membrane [21]. This promotes the expression of genes related to EMT activation. The fate of the cells is determined by the relationship between β-catenin and E-cadherin, which indicate the core driver for the canonical Wnt pathway and epithelial homeostasis maintainer, respectively. The over-expression of E-cadherin sequesters β-catenin and maintains its association with the cell membrane, preventing it from acting as a transcription driver. The dissolution of the adherens junctions from E-cadherin results in the translocation of β-catenin into the nucleus [22]. This step results in the activation of Snail expression, which suppresses the inhibition of epithelial-related gene transcription. E-cadherin decreases the NF-κB activity; this helps maintain the epithelial phenotype via the impairment of Snail1 activation and the induction of ZEB1 genes [23]. The canonical WNT signaling induces the formation and proliferation of neural crest precursors, wound healing, and the malignant progression of some types of cancer by inducing EMT [24]. NOTCH is another highly conserved signaling pathway that involves organ formation, tissue function, and tissue repair [25]. The binding of the NOTCH ligand to the receptor triggers receptor-mediated endocytosis, which triggers receptor formulation change and activation [26,27]. The NOTCH intracellular domain (NOTCH-ICD), which is the receptor activation form, translocates to the nucleus subsequently and binds to the transcriptional activator, resulting in target gene expression [26,27]. NOTCH signaling is active during the early stage of embryonic development; however, it is maintained at a low level during maturation. The loss of NOTCH or its receptor impairs SNAIL expression during the development of the heart, thereby affecting the endocardial EMT process. Abnormalities in the active NOTCH signaling pathway also play important roles in the progression of malignant tumors such as lung, breast, hepatocellular, ovarian, and colorectal cancers [28]. EMT markers, along with NOTCH pathway expression products, are expressed at the outer edge of the tumor, indicating the role of NOTCH in regulating EMT [29]. In addition, cross-talk between these signaling pathways can also induce EMT. Notably, miRNA, lincRNA, and epigenetic regulation are also involved in the EMT process.

Tumor cell EMT occurs due to the activation of the EMT signaling pathway in response to tumor microenvironment stress, such as hypoxia, low pH, immune responses, mechanical stress, and antitumor drugs. EMT endows cancer cells with several traits that facilitate progression malignancy, such as increased invasiveness and metastasis, stem-like properties, immunosuppressive effects, and resistance to antitumor drugs.

## 3. EMT and Metastasis

EMT is the initial and critical step of cancer metastasis that facilitates the progression of malignant cancers. However, EMT is an advanced event that may occur in precancerous stages. Rhim et al. tracked pancreatic cancer cells invading the bloodstream before malignancy in a mouse model of pancreatic cancer, indicating that EMT occurs during the early stage [30]. This is consistent with the clinical findings of some patients with one or more metastases, where the primary focus is not evident.

EMT is a focal event, rather than a global event, that occurs in neoplastic cells that have acquired genetic and epigenetic alterations in response to EMT stress [11]. Malik demonstrated that EMT-related protein expression was markedly elevated in the peripheral leading edge in NSCLC [31]. EMT is an indispensable driving force that promotes tumor progression in most types of cancer; however, it is unclear whether different types of tumors affect the location and degree of EMT occurrence.

Cancer cells acquire the ability to migrate following EMT; this ability is related to their partial or total mesenchymal phenotype. Migrating cancer cells invade microvessels and major blood vessels in the form of single cells or cell clusters known as circulating tumor cells (CTCs) [32,33]. These CTCs associate with platelets to survive and spread to distant tissues via circulation. The microvessels capture the CTCs, and the captured cancer cells are extravasated into the parenchyma of the tissues to form micrometastases. CTCs growing in the colonized niche are termed disseminated tumor cells (DTCs) [34]. However, most of these cells die owing to incongruous microenvironment conditions; some become dormant, and only a few survive. The surviving CTCs undergo MET, resulting in the restoration of the epithelial phenotype [35]. These cells form a macroscopic metastasis after colonization and reinitiate growth at the new site. Nevertheless, metastasis remains a dilemma in cancer research, with several unresolved problems. Notably, the entire metastatic cascade is extraordinarily inefficient, with only a small percentage of CTCs succeeding in forming macroscopic metastases [36]. This is similar to the hypothesis that only the right seeds can survive and germinate in the appropriate soil under the required conditions, such as sunlight, air, and humidity.

### 3.1. CTCs

As one of many seeds, how can a CTC stand out and become the right seed? CTCs present features of EMT and genotypes derived from primary cancers. CTCs express epithelial and mesenchymal markers, with significant heterogeneity being observed in different sources [37]. Nicole et al. used a lineage-labeled mouse model of pancreatic ductal adenocarcinoma to study EMT in vivo and revealed that most tumors exhibit a partial EMT phenotype and migrate as cell clusters rather than in the single cell migration pattern [38]. Furthermore, their study revealed that different EMT processes were correlated with tumor subtypes, resulting in distinct cell migration patterns. Partial EMT is usually associated with well-differentiated transcriptional subtypes, mainly disseminating in CTC clusters [38]. In contrast, complete EMT is strongly associated with poorly differentiated transcriptional subtypes, mainly disseminating in solitary CTCs [38]. CTCs exhibit genotypic characteristics of the primary tumors and relevance for metastasis potential; thus, they have been used to predict the prognosis, progression, and drug sensitivity of different types of cancer. Patients with refractory symptoms present with more mesenchymal-like CTCs and fewer epithelial-like phenotypes compared with patients who respond to treatment [39]. This finding indicates that in addition to the number change, the CTC phenotype changes before and after treatment in response to treatment. CTCs enter the bloodstream in the form of single CTCs (single cells) or CTC clusters (≥2 cells) and face several threats, including anoikis, the physical stress resulting from fluid shear and the killing of immune cells [18,40]. In contrast to single CTCs, CTC clusters obtained from partial EMT express cell–cell adhesion proteins, such as E-cadherin, desmosome, and hemidesmosome, are more resistant to anoikis, and protect against shear stress [18,40]. EMT plasticity enhances the ability of CTCs to resist fluid shear forces. CTC clusters can pass through tiny capillaries and decrease shear stress via reorganization, as observed in the zebrafish model [41]. The adherence of CTCs with platelets, neutrophilic granulocytes, or cancer-associated fibroblasts (CAF) within the bloodstream results in the formation of heterotypic CTC clusters, which prevent recognition by immune cells and inhibit natural killer (NK) cell activity by decreasing the activating immunoreceptor NKG2D on NK cells [42]. In addition, the enhanced expression of immune checkpoint proteins, such as PD-L1, in CTCs induces immunosuppression. Thus, CTC clusters, which are associated with survival advantages, are more likely to be the right seed than solitary CTCs.

### 3.2. Organotaxis Metastasis

The metastasis site of tumor cells from different sources is not random, i.e., these cells exhibit organ-specific preference or “organotropism”. The liver, lung, brain, and bone are the most common sites of metastasis in patients with lung cancer. The liver is one of the favored sites for distant metastasis for solid tumors. It receives a dual blood supply from the hepatic portal vein and hepatic arteries. Moreover, the sinusoid blood pressure gradient in the liver is significantly lower. This unique circulation feature allows access to the CTCs and facilitates their attachment to the sinusoidal endothelium for seeding [43]. Furthermore, compared with the well-organized endothelial wall and basement membrane in other organs, the fenestrated endothelial structure of the liver sinusoid is more permissive to extravasation [44]. The lung is another frequent site of metastasis in patients with different types of cancer. The physiology of the lung makes it ideal for the colonization and metastasis of CTCs. The broad surface area and numerous capillaries provide opportunities for the cancer cells to adhere, extravasate, and colonize [45]. The endothelial layer in the lung has tight junctions between endothelial cells and an intact basement membrane, representing a more restrictive barrier for extravasation than the liver [3]. The blood–brain barrier (BBB) is a continuous, non-fenestrated endothelial structure stitched together by tight junctions and supported by a basement membrane, astrocytes, and pericytes that protect the brain. The BBB prevents the circulating tumor cells from invading, making it the most difficult site to invade. Lung cancer is the most common source of brain metastasis (BM). In addition to target organ vasculature distribution and anatomical structure, organ-specific metastasis is also governed by the primary TME and metastatic organ microenvironment. EMT plays a crucial role in the progression of metastasis; however, it is unclear whether it affects the organotropic metastasis of tumors. Organotropic metastasis may be correlated with specific gene expression patterns of primary tumors. Qiang Li et al. reported that an increased risk of BM was related to APOBEC mutation and that TGFβ and EMT were upregulated in patients with high-level APOBEC mutational signatures [46]. ShengKai reported that mesothelin expression was significantly elevated in the serum and tumor tissue samples of patients with NSCLC who had BM [47]. Mesothelin expression promotes the tumorigenicity of human lung cancer by inducing the EMT and stemness of tumor cells. In addition, mesothelin significantly enhances the extravasation of NSCLC cells through the BBB to facilitate the incidence of BM. EMT-induced metastasis is often accompanied by tumor stromal degradation via the upregulation of matrix metalloproteases (MMPs) and plasminogen activators (PAs). Elevated MMP-9 levels have been observed in patients with NSCLC presenting with highly BM sub-clone cell lines compared with those in patients with no BM, suggesting a possible role in the migration to the CNS through the BBB [43]. Non-coding RNAs (ncRNAs) affect the metastasis of NSCLC by affecting EMT progression. Previous studies have explored the role of ncRNAs in EMT and lung cancer organ-specific metastasis. Many miRNAs in NSCLC, such as miR-21, have been correlated with the development of BM. miRNA-378, another oncogenic miRNA, is differentially expressed in patients with NSCLC presenting with BM [48]. MiRNA-378 has been linked to the activation of EMT and increased expression of metalloproteases (MMP-7 and MMP-9) and proangiogenic factors (VEGF), which facilitate the destruction of the BBB. Young Wha Koh et al. identified 25 miRNAs related to the BM of lung adenocarcinoma [49]. Notably, higher expression of the MET signature, TWIST, and vimentin was observed in this group. Distinct subtypes of cancer display significant variations in their organ specificity. Compared with squamous carcinoma, lung adenocarcinoma is more likely to metastasize to the brain and adrenal gland. EGFR, KRAS, BRAF, and ALK mutations are associated with a higher frequency of BM. In addition to the direct regulation of organotropism, EMT pathways may affect organ-specific metastasis via the regulation of the tumor immune microenvironment.

**Figure 1:**
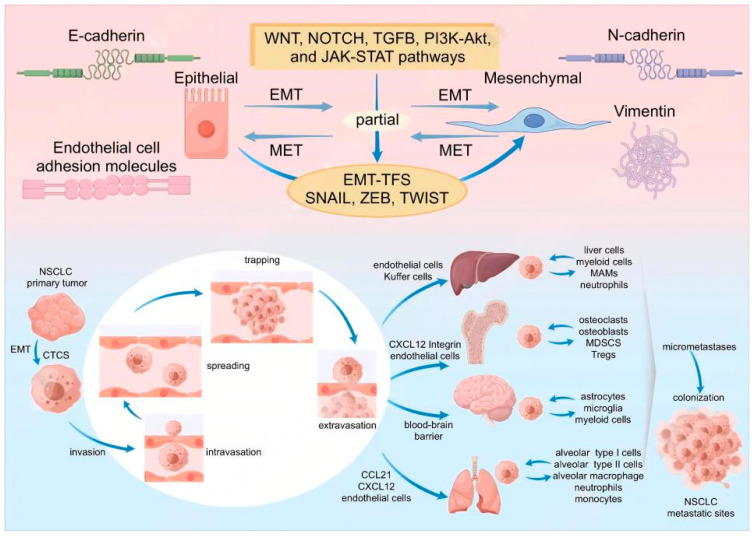
EMT and metastasis cascade in NSCLC.

In addition to the intrinsic mechanism in the cancer cells, immune cells and molecules in the TME promote EMT and organotropic metastasis in lung cancer cells directly and indirectly. EMT activator ZEB1 can suppress the EMT suppressor miR200, thereby relieving the miR200-mediated inhibition of PD-L1, an immune-checkpoint protein. This results in CD8+ T cell immunosuppression and metastasis [50]. Furthermore, EMT-induced modulation of E-cadherin and cell adhesion molecule 1 (CADM1) regulates NK cell-mediated metastasis-specific immunosurveillance [51]. Ke Xu identified three distinct cancer-associated fibroblasts (CAF) subpopulations in NSCLC that have different effects on metastatic sites: myofibroblastic CAF (myCAF), inflammatory CAF (iCAF), and antigen-presenting CAF (apCAF) [52]. Intercellular signaling network analysis revealed that apCAF is prevalent in patients with bone metastasis and that it activates the signaling pathways associated with cancer stemness. In contrast, iCAF is prevalent in patients with BM, and it activates invasion and metastasis-related molecules, such as MET hepatocyte growth factor [52]. The pre-metastatic niche (PMN) is the “congenial soil” favored by disseminated tumor cells (the seed) for colonization and outgrowth. Lung cancer is one of the types of cancer that frequently metastasizes to bones. Almost all important EMT regulators have been identified in the bone microenvironment facilitating bone metastasis formation, including hypoxia, various growth factors (TGFβ, epithelial growth factors, vascular endothelial growth factor, insulin-like growth factors, platelet-derived growth factor, and parathyroid hormone-related protein), cytokines (IL-1, 6, 8, and 11), and other signaling molecules (including integrins, MMPs, notch, Wnt, hedgehog signaling, and bone morphogenetic proteins [BMP] signaling pathways) [53]. The establishment of supportive pre-metastatic niches aids the survival and colonization of disseminated metastatic cells. Several studies have investigated the role of EMT in the formation of PMN. PMN comprises a series of processes, including the recognition of tumor-derived exosomes or tumor-shed extracellular vesicles (TDEVs) by the target organ resident cells. The site-specific spread of metastatic foci observed in solid tumors may be attributed to the chemokine secretion signature of a given tissue combined with the selective expression of the matched receptors by tumor cell clones. Chemokine–chemokine receptor axes established between tumor cells and specific organ sites facilitate metastatic microenvironment matrix remodeling as well as immunocyte recruitment [54,55]. Furthermore, these receptor axes, as critical communication bridges between tumor cells and stromal cells, enable the targeted migration of tumor cells to specific organ sites. Chemokine receptor C-X-C chemokine receptor 4 (CXCR4) and its ligand C-X-C motif chemokine ligand 12 (CXCL12) promote the metastatic potential of NSCLC in vitro and in vivo [56]. The overexpression of CCR7 in primary lung cancer specimens is a predictor of significant lymphotropic behavior. CXCR4 is aberrantly overexpressed in several types of cancer, including NSCLC. The CXCL12 overexpressed in some tissues and organs is a key niche factor that nurtures the pre-metastatic niches and recruits tumor cells to these niches, thereby fostering cancer cell aggression and metastatic capabilities [57]. The activation of the CXCL12–CXCR4 signaling axis promotes EMT and the mobilization of cancer stem/progenitor cells to pre-metastatic niches, thereby facilitating organ-specific metastasis. CXCL12/CXCR4 signaling stimulates EMT via the MED/ERK and PI3K/AKT signaling pathways in patients with sacral chondrosarcoma and the Wnt/β-catenin signaling pathway in patients with colorectal cancer [58]. Lunqing Wang reported the overexpression of CXCR4 protein in patients with BM compared with that in those with other organ metastases or without metastases, indicating that high-level CXCR4 expression is correlated with brain-specific metastasis in patients with NSCLC [59]. Moreover, the elevated expression of CXCR4 is especially primed to utilize the physiological survival signals in the bone marrow, increasing the probability of establishing overt metastasis in the future. Shuai Han demonstrated that elevated chemokine ligand 7 (CCL7) expression is associated with bone metastasis of NSCLC and that CCL7 may provoke NSCLC cell metastasis via EMT (mainly through receptor CCR3) [60]. In addition, the CCR6 receptor plays a role in the organ orientation of the development of metastases in lung cancer. Increased production of CCL20 in the adrenal glands may contribute to the selective recruitment of CCR6-expressing cancer cells in patients with lung cancer [61]. These tumor cells in the MME may culminate in the demise, dormancy, or successful establishment of a metastatic lesion after proliferation when disseminated tumor cells colonize the PMME of a specific organ (Figure 1).

## 4. EMT and TME

TME comprises immune cells, fibroblasts, extracellular matrix components, microvesicles, and various associated cytokines and chemokines, which act as the soil for tumor growth and the battlefield for killing tumor cells. The cytotoxic CD8+ T cells, NK cells, and classically activated M1 macrophages in TME have antitumor functions. However, compared with these immune cells, which promote an attack on the carcinoma cells, immunosuppressive regulatory T cells (Treg cells), alternatively activated M2 macrophages, MDSCs, and non-immune cells (such as CAFs) collectively inhibit T cell function and restrict T cell migration to the tumor nest, thereby aiding tumor progression [62]. EMT acts as a driver of metastasis and induces immunosuppression by affecting the TME. The survival of patients with many types of cancer, especially melanoma and lung, renal, head, neck, and liver cancers, has improved in recent years with the advances in the domain of cancer immunotherapies, especially ICIs, such as anti-PD-1 and anti-PD-L1. An increasing number of patients with cancer are receiving immunotherapeutic agents during the early and late stages of the disease. However, these patients present with different pathological types, genotypes, metastatic sites, and treatment backgrounds. Some patients do not respond well to immunotherapy, and the same tumor may exhibit inconsistent therapeutic responses in terms of primary and metastatic lesions or different metastatic sites. A greater understanding of the TME and relevant influencing factors will improve the effectiveness of immunotherapy and prevent the unnecessary implementation of immunotherapy. EMT accelerates cancer growth and metastasis by directly regulating cancer cells and reprogramming the immune response in the local TME. However, most cells, including immune cells and non-immune cells from the microenvironment, can secrete cytokines and chemokines to promote tumor cell EMT. This results in the formation of a vicious cycle that accelerates tumor progression. This review explored the molecular mechanism via which EMT promotes immunosuppression in the TME to identify strategies to improve the efficacy of immunotherapy by targeting EMT.

### 4.1. TME of NSCLC

TME is a double-edged sword for tumor cells. The cross-talk of tumor cells with immune cells and immune molecules determines whether the TME contributes to or inhibits tumor colonization and growth. The theory of cancer immunoediting highlights the role of TME in cancer progression, indicating that tumors adapt to survive via immune escape under selective pressure. TME can be divided into three immunotypes based on the distribution of cytotoxic lymphocytes (CTLs) and the expression of PD-L1 according to the classical theory. The immune desert type is characterized by the lack of immune cells within the TME [63]. This may be attributed to the repulsion or emigration of immune cells owing to the lack of attractive factors, resulting in no response to checkpoint inhibitors. Tumors classified as infiltrated–excluded are poorly immunogenic or cold, indicating poor response to ICIs. They are characterized by a relative lack of CTLs in the tumor core despite the presence of immune cells owing to the inhibitory matrix prevention. In contrast, tumors classified as infiltrated–inflamed are immunologically “hot” tumors that usually exhibit significantly higher responses to ICIs. They are characterized by the presence of high infiltration of CTLs and the expression of PD-1 and high expression of PD-L1 in the tumor and myeloid cells and are generally present at the invasive tumor margin or in the stroma, indicating enhanced inflammation. Furthermore, they display tertiary lymphoid structures (TLSs) and comprise many immune cells wherein lymphoid recruitment and immune activation occur. Tumor evolution occurs owing to the interaction between internal and external factors under pressure selection. Internal factors (such as different driver gene mutations and different metastatic sites) and external factors, such as treatment stress (including radiotherapy, chemotherapy, targeted therapy, and immunotherapy), reshape the TME landscape even among patients with the same pathological type of NSCLC.

EGFR, ALK, and KRAS are common driver gene mutations observed in patients with lung cancer. Compared with EGFR-wild NSCLC, EGFR-mutant NSCLC exhibits an immunosuppressive TME with inactive TILs. The EGFR signal upregulates the expression of PD-L1 via the PI3K-AKT, RAS-RAF-MEK-ERK, JAK-STAT3, and PI3K/AKT pathways, which further inhibit the CD8+ T cells [64]. The downregulation of CXC-chemokine ligand 10 (CXCL10) results in the downregulation of the recruitment of CD8+ T cells [64]. The EGFR signal upregulates CXCL22 and CC-chemokine ligand 5 (CCL5), resulting in an increase in the Treg cell levels. In addition, the interaction of various components in TME aggravates immunosuppression by inhibiting the function of CD8+ T cells and NK cells. Anaplastic lymphoma kinase (ALK) rearrangements account for approximately 5% of cases of NSCLCs, with EML4-ALK fusions being the most common. They exhibit specific TME features, such as reduced effector T cell levels and increased levels of immune checkpoint molecules, such as PD-1, LAG-3, and TIM-3 [65]. Short-term tyrosine inhibitor (TKI) treatment enhances T cell-mediated tumor clearance and reduces immunosuppressive M2 macrophage infiltration. In contrast, long-term TKI treatment fosters an immunosuppressive TME. ALK TKI-resistant tumors had low levels of CD8+ T cells and high expression of Treg cells [66].

The application of TKIs, especially those targeting EGFR and ALK mutations, modulates the tumor immune microenvironment in patients with NSCLC, contributing to the development of resistance. Short-term TKI treatment enhances T cell-mediated tumor clearance, reduces immunosuppressive M2 macrophage infiltration, and downregulates LAMC2 expression [67]. In contrast, long-term TKI treatment fosters an immunosuppressive TME. The TME of NSCLC with EGFR mutation changes following treatment with TKIs, which manifests as increased immunostimulation. The CD8+ T cell and Treg cell levels change with the duration of treatment and treatment sensitivity. The number of CD8+ T cells remains elevated initially; it remains unchanged thereafter. The infiltration of CD8+ T cells increases following TKI treatment in sensitive patients; in contrast, the infiltration decreases in patients with resistance. The infiltration and function of Treg cells are significantly reduced via the inhibition of the GSK-3b pathway along with the downregulation of CCL22 [68]. The number of Treg cells decreases and remains unchanged thereafter. The number of Treg cells decreases following TKI treatment in sensitive patients; in contrast, the infiltration is elevated in patients with resistance. The number of CD4+ T cells, along with the TNF-a and IL-2 expression, increases following TKI treatments in sensitive patients. A higher MHC class II expression in the infiltrated DCs has been observed in the TME [64]. ALK-targeted therapies elicit a stronger antitumor immune response than EGFR-targeted therapies [65]. The expression of PD-1 and other coinhibitory receptors, such as LAG3, TIM3, and TIGI, is a marker of T cell activation and exhaustion. The effector activity of the T cells is reduced in exhausted states owing to the low content of cytolytic factors. The findings of studies on PD-L1 expression in EGFR-mutant NSCLC populations have been controversial. Tang conducted an immunohistochemical analysis of the tissue samples of 170 patients with lung adenocarcinoma and demonstrated that PD-L1 overexpression is more likely to be correlated with EGFR mutation [69]. Several other preclinical studies have demonstrated a consistent correlation between EGFR mutation and PD-L1 expression. In contrast, Soo et al. conducted an immunohistochemical analysis of the tissue samples of 3969 patients from 18 studies and reported that patients with EGFR-mutant NSCLCs are less likely to be PD-L1-positive than those with wild-type EGFR tumors [70]. In addition, analyzing the mRNA and protein levels of PD-L1 in the Cancer Genome Atlas (TCGA) and internal database (Guangdong Lung Cancer Institute; GLCI) revealed lower PD-L1 mRNA and PD-L1 protein expression in the EGFR-mutated NSCLC samples compared with that in the wild-type tumor samples [71]. This discrepancy may be attributed to the heterogeneity of the study population. Some concomitant mutations, such as KRAS, TP53, and STK11, significantly affected the PD-L1 expression.

Therefore, the TME immunotype cannot be summarized using this classification alone owing to its dynamic and complex characteristics. In addition to driver gene mutations, different metastatic sites also affect the TME characteristics, which comprises complex interactions between tumor, tissue-resident, and immune cells in metastasis niches. In addition to the lungs, the liver, bones, and brain are the most common metastatic sites in patients with NSCLC. The liver is considered a less immunogenic organ. The cancer cells are exposed to a complex and distinct microenvironment on entering the liver, which comprises dendritic cells, Kupffer cells, and sinusoidal vessels, that enable immunosuppressive functions to facilitate cancer progression. Kupffer cells, the largest tissue-resident macrophage population, mainly mediate the immunosuppressive liver microenvironment via the expression of CTLA-4, PD-L1, TGF-β, or IL-10, and low expression of co-stimulatory molecules (CD80 and CD86) [72], thereby activating Treg cells. Treg cells suppress normal self-antigen and antitumor immune responses by reducing the infiltration of CD8+ T cells at the margins of distant tumors. Genomic landscape profiling reveals similar somatic mutations, tumor mutational burden, and neoantigen numbers in the pulmonary and liver metastases of patients with NSCLC. In contrast, the microenvironment of the liver metastases has lower levels of immune activation and infiltration than the primary lesion of the lung cancer [73]. The presence of liver metastases may also modulate the systemic anti-tumor immune response at other sites of metastasis. Tumeh et al. revealed that the CD8+ T cell levels were lower at distant sites in patients with liver metastases compared with those in patients without liver metastases [74]. The brain microenvironment is immunologically cold at all times. The BBB hinders the infiltration of tumor cells and immune cells [75]. Following invasion, the intracranial microenvironment is altered, with a disrupted BBB, characterized by the widespread infiltration of lymphocytes and phenotypical modification of the brain cells. This TME of BM comprises tumor cells and stromal components, such as fibroblasts, astrocytes, and an array of immune cells (including microglia, macrophages, and lymphocytes). Reactive astrocytes and tumor-associated macrophages play a paramount role in the BM of patients with NSCLC [76]. Furthermore, they promote tumor progression and immune evasion in the BM. The microenvironment of BM is immunosuppressed compared with that of the primary tumor in terms of the suppression of immune-related signaling pathways, such as diminished expression of immune checkpoints, reduced infiltration of CD8+ T cells and cytotoxic lymphocytes, and a higher proportion of immunosuppressive M2 macrophages, owing to the presence of similar somatic hot spot mutations [77]. Bones are another common metastatic site for lung cancer. Bone metastases with a unique tumor immune landscape (characterized by an increased infiltration of fibroblasts and stromal cells compared with that in other metastases, such as the liver and brain) harbor specific immune cells and tumor cells. An increase in the tumor expression of PD-L1 and VCAM-1 compared with that in other metastases (such as the liver and brain) has also been observed. The upregulation of the RANKL pathway leads to an increase in the number of osteoclasts, which subsequently increases osteoclast-driven bone resorption in bone metastases [78]. In addition, RTs generally demonstrate significantly lower CD8+ cytotoxic T lymphocyte (CTL) density and lower CD68+ macrophage density, with a loss of tertiary lymphoid structure (TLS) in patients with NSCLC who have received platinum-based chemotherapy postoperatively. This may potentially impact the therapeutic benefits of immunotherapy [9].

### 4.2. The Effect of EMT on TME

The acquired mesenchymal or quasi-mesenchymal traits of the cancer cells that undergo EMT can modulate the immune microenvironment partly by modifying the number and function of innate and adaptive immune cells to facilitate cancer progression. The resulting changes in immune cells will further induce EMT in tumor cells, resulting in the formation of a vicious cycle that exacerbates tumor growth. The cytotoxic CD8+ T cells, NK cells, and classically activated M1 macrophages exert antitumor functions in the TME. In contrast, some populations of immune cells, such as immunosuppressive Treg cells, alternately activate M2 macrophages. MDSCs suppress the function of T cells and NK cells. EMT-induced immunosuppressive effects, which have been observed in many types of cancer, indicate poor response to immunotherapy. Kudo-saido reported that the activation of EMT can induce the formation of immunosuppressive Treg cells and resistance to checkpoint blockade therapies in patients with melanoma [9]. Breast tumors derived from more epithelial carcinoma cell lines express high levels of MHC-I and low levels of PD-L1. Furthermore, the TME is infiltrated with CD8+ T cells and M1 (antitumor) macrophages. In contrast, tumors derived from more mesenchymal carcinoma cell lines exhibiting EMT markers express low levels of MHC-I, high levels of PD-L1, infiltration of regulatory T cells and M2 (protumor) macrophages, and exhausted CD8+ T cells in the microenvironment [79]. Epithelial tumors are more susceptible to immunotherapy than mesenchymal tumors. An integrated gene expression analysis of three independent datasets revealed that in patients with NSCLC, lung adenocarcinoma with a “mesenchymal” phenotype exhibits distinct tumor microenvironment changes. The EMT status is strongly associated with an increased population of co-existent CD4+, Foxp3+, Tregs, and CD3+ T cells, as well as the elevation of multiple immune checkpoint molecules [64]. These findings indicate the role of EMT in reprogramming the immune response in the local TME.

The cancer-immunity cycle refers to the anti-cancer immune responses involving a series of events, including the tumor antigen release, antigen presentation, T cell activation, trafficking, and infiltration of T cells into tumors, and recognition and killing of tumor cells that enhance and expand over time through continuous recognition and memory of tumor antigens [63]. The TME is the starting and endpoint of the tumor immune cycle. T cell migration through the tumor stroma, interaction with intra-tumoral immune cells, and persistent functioning within the TME hold a central role in the cancer-immunity cycle and a microcosm of what occurs beyond the tumor. The active and effective anti-tumor immune response requires the infiltration of a certain proportion of T cells with normal functioning into the TME. Thus, tumor cells undergoing EMT disturb the cancer-immunity cycle by inhibiting the infiltration of functional T cells or inducing the infiltration of immunosuppressive cells, such as polymorphonuclear MDSCs, myeloid cells, mast cells, and natural CD4+ CD25-Tregs, but not necessarily accompanied by reduced tumor infiltration of immune cells, called immune rejection and immune bias mainly [62].

EMT imparts phenotypic changes to cancer cells; however, it can reduce the expression of the tumor antigen, which protects the cells from antigen-specific T-cell killing. Reduced antigen presentation may occur due to the inhibition of antigen processing. The immunoproteasome generates antigenic peptides that bind to human leukocyte antigen (HLA)-I molecules that facilitate recognition by CD8+ T cells. Immunoproteasome deficiency is observed in patients with NSCLC with a mesenchymal phenotype [80]. Thus, EMT is involved in the downregulation of antigenic peptide presentation molecules for immune escape. EMT also enhances the resistance of tumor cells to the cytotoxic effects of cell lysis. Tumor cells undergoing TF brachyury-mediated EMT to overexpress transmembrane glycoprotein mucin-1 (MUC-1) will exhibit reduced susceptibility to CTL killing even with normal levels of HLA class I, antigenic peptides, and components of the antigen presentation [81].

The activation of EMT-TFs is the core driver for EMT. Snail1, a key EMT-TF, is involved in tumor immunosuppression and immune evasion. It recruits immunosuppressive cells into the TME via chemokines and induces T-cell exhaustion in patients with melanoma. A histochemical staining of 477 lung adenocarcinoma specimens revealed a positive correlation between the expression of PD-L1 and Snail1. Snail1 also increased intra-tumoral C-X-C chemokine ligand 2 (CXCL2) secretion and neutrophil infiltration in a mouse model of lung cancer [82]. Twist1, another important EMT-TF, regulated the TME via immune infiltration in 44 types of cancer, including NSCLC, in the study by Wang et al. The findings of these studies indicate that fibroblasts, which exhibit the potential to suppress and compartmentalize CD8+ T cells and other immunocytes, are one of the most important infiltrating cells indicating poor prognosis [83]. Furthermore, fibroblasts have significant positive correlations with immune checkpoint expressions across pan-cancers. These findings also suggest that Twist1 is positively correlated with immunomodulators, such as CCL3, CCL4, CCL11, CCL26, CCL8, CXCL8, CD276, CXCR4, and TNFSF18 [84]. ZEB1, another critical inducer of EMT, can recruit immunosuppressive cells (such as Tregs, MDSCs, M2 macrophages, and neutrophils) to participate in the formation of the TME via cytokines, chemokines, and their receptors. Moreover, ZEB1 directly or indirectly regulates PD-L1 to aggravate the immunosuppressive TME. Goswami et al. demonstrated that the miR-200/ZEB1 axis can indirectly regulate PD-L1 transcription and that it is an independent regulatory factor of PD-L1 [85]. PD-L1 expression is significantly higher in patients with mesenchymal and epithelial–mesenchymal phenotypes in pulmonary adenocarcinoma. Sandra Ortiz-Cuaran et al. reported that the EMT-inducer ZEB1 induces the immunosuppressive environment in NSCLC by impacting CD70 expression and fostering increased activity of the CD70 promoter, a regulatory ligand from the tumor necrosis factor ligand family, which is associated with decreased CD3+ and CD8+ T cell infiltration and increased T cell exhaustion [6]. Hypoxia is an important factor that induces EMT. The effect of the participation of soluble molecules, such as cytokines and chemokines, in immune circulation can be negative or positive. Some of these molecules may be associated with two-sided effects. The effects of tumor cells with EMT on the immune system are largely mediated by cytokines and chemokines secreted from them. Lung adenocarcinoma exposed to hypoxia exhibits resistance to killing by CTLs and NK cells via defective immune synapse signaling. In addition, hypoxia regulates the activities of MDSCs and recruits them to local tumors via CCL26 or CD39L1 [86]. The acquisition of the EMT phenotype, which is characterized by dramatic morphologic changes and actin cytoskeleton remodeling in human breast cancer cells, results in attenuation of the formation of an immunologic synapse with CTLs along with the induction of autophagy in the target cells to inhibit CTL-mediated cell lysis. TGF, a vital EMT inducer, can downregulate the expression of TNFa and IL12 via TGFb/Snail signaling, thereby promoting the differentiation of unactivated macrophages into TAM-like phenotypes [87]. TGFβ also upregulates the expression of PD-L1 via the activation of the PI3K/Akt pathway. In addition, TGF-induced EMT leads to the development of resistance to complement-dependent cytotoxicity by inhibiting the formation of membrane attack complexes. The overall proportion and function of T cells within the TME also affect the effectivity of tumor immunity. The increased surface expression of inhibitory receptors (CTLA-4, PD-1, TIM-3, LAG-3, and 2B4) on T cells; T cell effector function loss, such as the production of cytokines IFNγ, IL-2, and TNFα; and the loss of T cell proliferative capacity will result in the dysfunction of T cells. Immune checkpoint molecules act as “coolant” to activate an immune response, in addition to playing an immunosuppressive role. Chao et al. observed PD-L1 positivity in adenocarcinoma in situ, minimally invasive adenocarcinoma, and invasive adenocarcinoma, indicating that immunoediting commences before invasion [88]. The anti-tumor response is weakened by the interaction between the immune-checkpoint proteins expressed on the tumor cells and their ligands expressed on the T cell surface. Yanyan Lou revealed that distinct TME changes are observed in lung adenocarcinoma with a “mesenchymal” phenotype. In contrast to lung adenocarcinoma with an “epithelial” phenotype, it constitutes endogenous immune activation, such as elevated levels of the immune co-stimulatory molecules (i.e., IFN-γand CXCL10), along with the simultaneous elevation of multiple immune checkpoint molecules (e.g., elevated PD-1 and PD-L1) [89].

In addition to directly affecting the immune cells within the TME, tumor cells that induce EMT also act on the tumor immune microenvironment indirectly by influencing the non-immune stromal components to regulate TME immune responses. Approximately 40% of patients with NSCLC present with infiltrated-excluded immune subtype, which is characterized by the presence of densely arrayed fibrotic nets present circumferentially around tumors with CD8 T cells and CAFs. The stromal niche and CAFs along the tumor impede the infiltration of CD8+ T cells into tumors, thereby hindering the response to checkpoint blockade inhibitors. The ECM is produced by CAFs that can be regulated by TGFβ signaling. The activation of the TGFβ pathway plays a critical role in triggering and maintaining EMT. However, the development of CAFs is dependent on fibroblast-intrinsic TGFβ signaling. The CAF-deposited matrix is associated with reduced lung tumor infiltration by T cells and DCs, as well as alterations in the TAM states. Thus, the inhibition of EMT via the blockade of TGFβ signaling can alter the stromal architecture and permit T cell entry in preclinical models.

As mentioned previously, different metastatic sites of NSCLC vary in terms of tumor immune spectrum and immunotherapy response. The local heterogeneity of the tumor immune landscapes could be partly attributed to the tumor epithelial–mesenchymal plasticity. Primary carcinoma cells undergoing heterogeneous EMT produce and secrete various factors, such as exosomes carrying specific miRNAs, integrins, inflammatory cytokines, growth factors, and extracellular matrix enzymes, thereby inducing the formation of a metastatic organ microenvironment suitable for metastatic tumor growth via ECM remodeling and the regulation of immune cells and metastatic site resident cells. The majority of important EMT regulators, such as hypoxia, TGFβ, epithelial growth factors, vascular endothelial growth factor, and EMT signaling molecules (including integrins, MMPs, notch, Wnt, hedgehog signaling, and BMP signaling pathways) have been detected in the bone microenvironment. All immune cells are derived from the bone marrow, thereby creating a close relationship between the bone environment and the immune system. NSCLC bone metastases tend to transform the bone microenvironment into becoming more osteoclast-dominant to facilitate increased bone resorption, which is related to the increased activation of the RANKL pathway, TGFβ, and IGF. The interactions between T cells and osteoclast precursors through reciprocal CD137/CD137L and RANK/RANKL regulate bone absorption [90]. MDSCs are progenitors of the osteoclast precursors; consequently, their number is increased in bone metastases, which greatly inhibits the anti-tumor T cell response. With the growth of tumors, a local hypoxic environment prompts tumor cells to secrete factors and extracellular vesicles to attract MDSCs from bone marrow to further induce an immunosuppressive environment.

STAT3 is another key stimulator for EMT. BM from different primary tumor sources can be reduced by experimentally blocking STAT3 signaling in reactive astrocytes. The STAT3-positive reactive astrocytes promote immunosuppressive TME by suppressing the activation of CD8+ T cells and promoting the expansion of CD74+ microglial/macrophages. This is beneficial for metastatic tumor growth in the brain [91].

In summary, tumor EMT exerts immunosuppressive effects on the microenvironment via different mechanisms in patients with NSCLC, regardless of the primary lesion or metastasis. Thus, it is an opportunity and a challenge for immunotherapy.

## 5. Targeting EMT in NSCLC: Therapeutic Opportunities and Challenges

**Figure 2:**
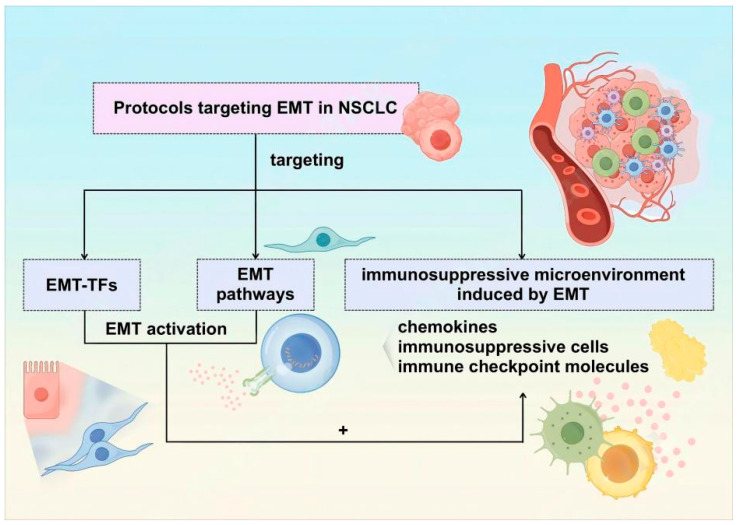
Available therapeutic options for targeting EMT.

The deepening of our understanding of EMT has led to the realization of the important role it plays in physiological and pathological processes, especially in cancer. This review summarized the molecular and cellular mechanisms through which EMT promotes tumor progression, remodels the tumor immune microenvironment, and induces drug resistance. The inhibition of EMT can inhibit cancer progression, increase treatment sensitivity, and delay the development of drug resistance in multiple ways. EMT-TFs directly activate EMT. Drugs targeting EMT-TFs have been developed and used in clinical trials. Brachyury, an EMT transcription factor, plays an important role in the differentiation of embryonic notochords. The Brachyury gene is closely related to the occurrence, development, and sensitivity to radiotherapy and chemotherapy of epithelial-derived tumors, such as chordoma, breast cancer, lung cancer, and prostate cancer. The inhibition of Brachyury gene expression yielded a T cell response in a Phase I study (NCT01519817); however, the NCT02383498 chordoma study revealed no benefit [92]. EMT-related intracellular signaling pathways cooperate to induce the expression of EMT-TFs that act pleiotropically to induce EMT. The TGFβ pathway serves as an important EMT-related pathway and confers invasive, metastatic, and therapeutic resistance to cancer cells. In addition, it also contributes to the formation of immunosuppressive TME. A Phase II study on galunisertib monotherapy, a small molecule inhibitor of TGFβ type I receptor, yielded promising clinical data regarding liver cancer, with a median OS of 16.8 months in patients with baseline AFP < 1.5 × ULN [93]. In addition, among the 15 patients with advanced hepatocellular cancer who were treated with a combination of galunisertib and SBRT, two patients achieved PR and six patients had SD (57% DCR). Administering a combination of galunisertib and neoadjuvant chemoradiotherapy to patients with locally advanced rectal cancer increased the CR rate to 32%, indicating that TGFβ inhibition elicits a response to chemoradiotherapy [94]. Vactosertib, another novel inhibitor of the TGFβ type I receptor, is also being recruited for clinical studies owing to the efficacy observed in a mouse model of breast cancer [95]. A Phase II clinical trial of AB-16B5, a potent inhibitor of EMT, in combination with docetaxel (NCT04364620) is planned for patients with NSCLC showing progression after chemotherapy or PD-1 therapy. ICI treatment failure and tumor progression can be attributed to the immunosuppression within the TME induced by EMT, which is characterized by the intra-tumoral infiltration of immunosuppressive cells and the expression of immune checkpoint molecules [9]. Thus, the combined targeting of immunosuppressive cells and cytokines can improve the efficacy of ICIs in EMT-transformed tumors. The combination of anti-PD-1 antibody and anti-CD 25 antibody (RG6292) [96], which aims to induce Treg cell depletion, induced tumor growth inhibition in mice models. TAM, another immunosuppressive cell, can be a potential target. Clinical trials have used anti-CCL2 or anti-CCR2 antibody to inhibit CCL2 chemotactic TAMs in the local tumor microenvironment via CCR2 in addition to targeting MDSC drugs (such as all-trans retinoic acid), some chemotherapies (such as 5-fluorouracil and gemcitabine), and CXCR2 chemokine or its ligands (such as CXCL1 and CXCL2 blocking). The CXCR2 antagonist SB265610 suppresses MDSC migration and inhibits the progression of Snail-overexpressing ovarian tumors [97]. The combination of ICIs and CXCR2 antagonists inhibits the enrichment of MDSCs in the TME and improves the potency of CTL to further improve the anti-tumor effect. This model has been used in clinical studies. Comprehensive therapy targeting EMT and immunosuppression is the focus of current research. Bintrafusp alfa (M7824), a TGFβ/PD-L1 dual-targeting fusion drug, simultaneously blocks TGFβ and PD-L1 immunosuppressive pathways in the TME [98]. This drug can inhibit TGFβ-mediated EMT in vivo and in vitro. Furthermore, compared with PD-L1 monotherapy, this drug increased intra-tumoral CTL infiltration and decreased the number of NK cells and MDSC cells in a mouse model of breast cancer. The effects of this drug have been clinically studied in multiple solid tumors. However, a Phase III clinical trial of bintrafusp alfa versus Keytruda in lung cancer was terminated early owing to the lack of efficacy [99]. EMT is a good target owing to its multi-effect biological role in the occurrence, development, metastasis, and drug resistance of malignant tumors; however, it is also challenging in the implementation process (Figure 2).

EMT enables tumor cells to invade surrounding tissues and disseminate to distant sites. Anti-tumor immunity is an important factor that prevents tumor metastasis and successful colonization. However, tumors undergoing EMT can recruit immunosuppressive cells and trigger T cell depletion by inducing immune checkpoint expressions, thereby triggering immunosuppression in the TME. This promotes the dissemination and colonization of tumor cells. In addition, immunosuppressive TME can act on tumor cells to trigger EMT. This positive feedback regulation between the EMT-phenotypic tumor cells and the immune microenvironment provides a constant impetus for the progression of malignant tumors. EMT also contributes to drug resistance. The pleiotropic biological function of EMT makes it a good therapeutic target for malignant tumors. However, the implementation process is full of difficulties and challenges. First, different incentives, different stages of tumor progression, and different sites of tumorigenesis can trigger the activation of different EMT-related pathways. This can promote the expression of heterogeneous EMT-TFs, resulting in EMT initiation. Therefore, using the heterogeneity of the EMT activation process to determine the specific targets to be selected is not feasible. Second, tumor cells show a series of phenotypes ranging from complete epithelium to complete mesenchyme following EMT, which induces immunosuppression to different degrees via various mechanisms. In addition, the dynamic changes in the tumor cells also exert a dynamic effect on the immune microenvironment during tumor progression. This makes it impossible to only produce a transient effect by targeting a single type of immunosuppressive cell or immune molecule. The therapy targeting EMT has received keen attention in recent years. The candidate drugs simultaneously target multiple activators of the EMT pathway, such as targeting immune checkpoints and immunosuppressive cells in the TME transformed by EMT or targeting multiple targets (such as immune checkpoints and immune microenvironment inhibitory cytokines). These drugs have been tested in preclinical and clinical studies, but have not achieved satisfactory results. Systematic studies will provide a more precise picture of TME and offer novel approaches for therapeutic exploitation. The feedback loop between EMT and immune suppression should be further investigated to aid in the development of efficient strategies for cancer treatment.

## 6. Conclusions

EMT has been a driving force for tumor occurrence, development and evolution. The role of EMT in promoting tumor metastasis and inducing immunosuppression makes it a promising target for tumor therapies. However, challenges always go hand in hand with opportunities. The problems, such as the differences in EMT expression profiles in the same site at different disease stages, the dynamic evolution of cells among different EMT states, and the heterogeneity of EMT profiles between the primary site and metastatic site have been unsolved. In the future, the better understanding of EMT programme may help clarify them and explore the ideal targets for EMT. 

## Data Availability

Not applicable.
